# Investigation of Ultrasound Transmit–Receive Sequence That Enables Both High-Frame-Rate Vascular Wall Velocity Estimation and High-Contrast B-Mode Images

**DOI:** 10.3390/s25082441

**Published:** 2025-04-12

**Authors:** Hitoshi Hirano, Rikuto Suzuki, Masaaki Omura, Ryo Nagaoka, Kozue Saito, Hideyuki Hasegawa

**Affiliations:** 1 Graduate School of Science and Engineering, University of Toyama, Toyama 930-8555, Japan; 2 Faculty of Engineering, University of Toyama, Toyama 930-8555, Japan; momura@eng.u-toyama.ac.jp (M.O.);; 3 Department of Neurology, Stroke Center, Nara Medical University, Nara 634-8522, Japan

**Keywords:** ultrasound, phase-sensitive motion estimator, multi-line transmission, pulse inversion

## Abstract

In this study, we designed an ultrasound transmit–receive sequence to achieve high-frame-rate vascular wall velocity estimation and high-contrast B-mode imaging. The proposed sequence extends conventional dual-transmission schemes by incorporating a third transmission with 180° phase inversion, enabling harmonic imaging via the pulse inversion (PI) method. To mitigate the frame rate reduction caused by the additional transmission, the number of simultaneously transmitted focused beams was increased from two to four, resulting in a frame rate of 231 Hz. A two-dimensional phase-sensitive motion estimator was employed for motion estimation. In vitro experiments using a chicken thigh moving in two dimensions yielded RMSE values of 3% (vertical) and 16% (horizontal). In vivo experiments on a human carotid artery demonstrated that the PI method achieved a lumen-to-tissue contrast improvement of 0.96 dB and reduced artifacts. Velocity estimation of the posterior vascular wall showed generally robust performance. These findings suggest that the proposed method has strong potential to improve atherosclerosis diagnostics by combining artifact-suppressed imaging with accurate motion analysis.

## 1. Introduction

Atherosclerosis is a major contributor to cardiovascular disease, highlighting the critical importance of early diagnosis and the accurate assessment of disease progression. Ultrasound imaging, as a non-invasive and real-time diagnostic modality, has been extensively utilized for vascular assessments. Diagnostic parameters include both imaging-based indicators—such as plaque characterization [1,2] and the carotid intima–media thickness (IMT) [3,4,5,6]—and dynamic indicators that evaluate vascular wall motion and blood flow dynamics. Integrating these indicators offers the potential for more precise and efficient atherosclerosis diagnosis and the monitoring of treatment outcomes.

Moreover, advancements in imaging techniques can reduce the time required for data acquisition, thereby lessening the physical and psychological burden on patients while streamlining the diagnostic workflow for clinicians. In imaging-based diagnostics, achieving high-contrast and artifact-free images remains a significant challenge. Ultrasound imaging often suffers from degradation caused by artifacts, particularly those generated from side or grating lobes [7,8], which can obscure critical features such as vascular lumen boundaries.

Tissue harmonic imaging (THI) [9,10,11,12], which leverages harmonic components, has been proposed to address these limitations. Among the various methods employed in THI, the pulse inversion (PI) technique [13,14] is particularly effective in enhancing the spatial resolution and image contrast. The PI method is a key technique for harmonic imaging and is widely applied in both contrast harmonic imaging (CHI) [15,16] and THI. CHI employs microbubble contrast agents to exploit their nonlinear acoustic responses, while THI utilizes harmonic components generated through waveform distortion caused by the nonlinear propagation of ultrasound in tissue. However, the PI method requires multiple transmissions, which results in reduced frame rates—a critical limitation for applications requiring a high temporal resolution.

Dynamic indicators, such as vascular wall motion and the local pulse wave velocity (PWV) [17,18], are used to assess arterial stiffness, a key marker in atherosclerosis diagnosis. Various velocity estimation techniques—such as vector Doppler [19,20], speckle tracking [21,22], and two-dimensional (2D) phase-sensitive motion estimation [23]—have been proposed as angle-independent methods. Speckle tracking estimates the tissue displacement based on image pattern correlation but requires interpolation to capture small displacements that are below the sampling intervals of ultrasonic echo signals. In contrast, the 2D phase-sensitive motion estimator utilizes phase information to directly measure sub-sample displacements without interpolation.

High-frame-rate imaging using unfocused transmit beams, such as plane waves, has emerged as a technique to achieve frame rates of thousands of frames per second, and its benefits in cardiovascular applications have been demonstrated. However, it is difficult to use harmonic imaging with this method because the sound pressure of an unfocused transmit beam is significantly lower than that of a focused beam. Our research group has developed a transmit (Tx)–receive (Rx) sequence that employs focused Tx beams for the high-frame-rate acquisition of dynamic indicators, using multi-line transmission imaging (MLTI) [24] to estimate blood flow velocity vectors. However, this sequence was not designed to accommodate high-contrast imaging techniques, such as pulse inversion (PI), which are used for imaging-based indicators.

We propose an improved Tx–Rx sequence that integrates the previously developed MLTI sequence [24] with the PI method to enhance diagnostic imaging while maintaining a high frame rate. The proposed sequence introduces a third transmission with a 180° phase inversion for the PI method, following two same-phase transmissions from the same aperture. In this sequence, the two same-phase transmissions are still required because velocity estimation cannot be performed by calculating the phase shift between transmissions of opposite polarity. Additionally, the time interval between transmissions must be minimized to maintain the highest aliasing limit. Although the PI method has previously been applied to tissue motion estimation [25], the proposed sequence enables the simultaneous measurement of both blood flow and tissue motion, along with the high-contrast B-mode imaging of tissue—specifically, the arterial wall in this study. To compensate for the frame rate reduction caused by the additional transmission, the number of simultaneously transmitted focused beams can be increased. In the present study, an inverted emission was added as the third transmission to implement the traditional PI method. However, the proposed sequence can also be applied to other methods that require multiple transmissions.

In this study, the effectiveness of the proposed sequence for tissue motion estimation was evaluated through both in vitro and in vivo experiments. In the in vitro experiments, a chicken thigh was moved at a constant velocity using an automated stage to investigate the impact of PI-based B-mode imaging and the influence of the additional phase-inverted transmission on velocity estimation. In the in vivo experiments, human carotid artery data were acquired to evaluate the contrast between the vascular lumen and surrounding tissues, as well as the velocity estimation performance at the vascular walls in B-mode images.

## 2. Methods

### 2.1. Tx-Rx Sequence

**Figure 1:**
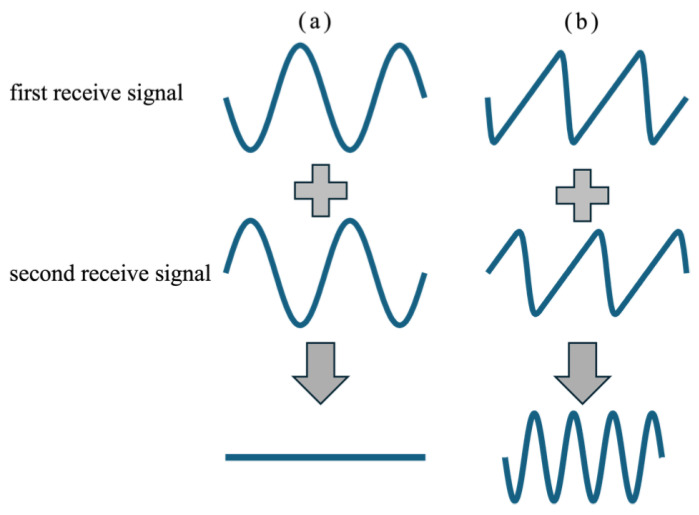
Schematic illustration of the pulse inversion (PI) method. (**a**) Summation of fundamental signals. (**b**) Summation of signals containing harmonics generated by nonlinear propagation.

Figure 1 provides an overview of the pulse inversion (PI) method. As illustrated in Figure 1a, when ultrasonic pulses propagate through tissue at low acoustic pressure, the propagation is nearly linear. However, as the acoustic pressure increases, tissue exhibits nonlinear responses, causing the ultrasonic signal to travel faster in regions of higher pressure and slower in regions of lower pressure [26,27]. As shown in Figure 1b, this results in a distorted received signal that contains harmonic components. The degree of distortion, or the extent of harmonic component generation, is determined by the nonlinear parameter B/A [28].

**Figure 2:**
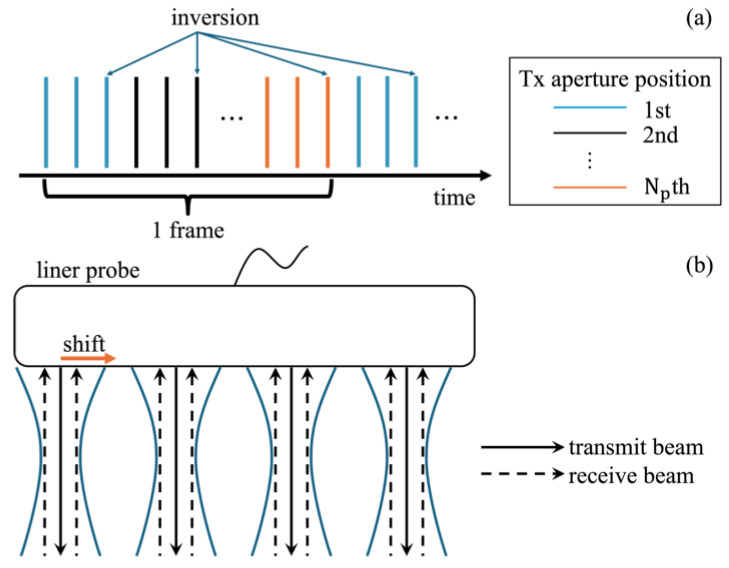
Illustrations of transmit–receive (Tx–Rx) sequences. (**a**) Transmit sequence. (**b**) Multi-line transmission and reception.

The repeated transmit (Tx) sequence used in this study is illustrated in Figure 2a. In this study, the Tx–receive (Rx) sequence using focused transmit beams from our previous work was extended to support the PI method. In the original sequence, the same Tx–Rx event is repeated twice at the same transmit aperture to maximize the aliasing limit of the Doppler method. For the PI method, a third transmission—a pulse with a phase shift of π relative to the previous two—was added at the same transmit aperture.

In this study, four focused beams were transmitted in parallel, as shown in Figure 2b. Each focused Tx beam was transmitted with 30 elements. The depth position of the transmit focal distance was set at 20 mm, and a Tukey window with a taper factor of 0.4 was used as the Tx apodization, based on our previous studies [24]. For echo reception, two steered receive beams were created at a position of 0.1 mm to the left and right from the center of the transmit beam, in addition to a non-steered beam for each Tx beam using DAS beamforming, as in Ref. [24]. The x- and z-axis spacings of the RF signals were set to 0.2 mm and 0.025 mm, respectively. These techniques generate eight receiving lines in one Tx–Rx event. In this study, the Tx aperture was moved at a pitch of 0.4 mm (2 elements) and the Tx–Rx event was repeated at 15 positions $N_{p}$. As a result, 4×2×15=120 receive lines (beamformed RF signals) were produced at each Rx steering angle in one frame. To generate a B-mode image, the beamformed RF signals obtained at three different steering angles were coherently averaged.

### 2.2. Basic Experimental Setup

A linear-array probe (UST-5412, Fujifilm, Tokyo, Japan) was connected to a custom-made ultrasound scanner (RSYS0016, Microsonic, Tokyo, Japan), and echo signals from the phantom were acquired from individual transducer elements at a sampling frequency of 31.25 MHz. Each element in the probe was excited with a two-cycle rectangular pulse at an amplitude of approximately 25 V at 4.8 MHz. The pulse repetition frequency was set to 10,417 Hz. The speed of sound used for receive beamforming was set to 1500 m/s, based on the water temperature of 26 °C.

**Figure 3:**
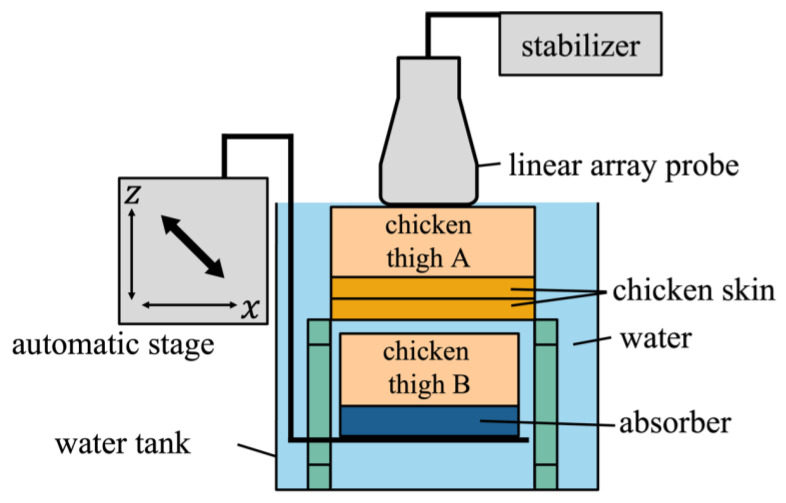
Illustration of basic experimental setup.

Figure 3 shows the experimental setup, in which an automated stage was moved and measured using an ultrasonic probe for velocity estimation. The chicken thigh and sound absorber were placed on a jig connected to the automated stage and moved at –1.0, –1.5, and –2.0 mm/s in both the axial (z) and lateral (x) directions. The chicken thigh and skin were fixed in front of the probe using metal wires, which were placed outside the imaging field of view. In the B-mode images, points of interest (POIs) were set on the chicken thigh, and velocity estimation was performed using the phase-sensitive two-dimensional (2D) motion estimator [23]. The averaged signal from the first and second transmissions at the same aperture was used for fundamental imaging, while the summed signal from the second and third transmissions was used for harmonic imaging.

**Figure 4:**
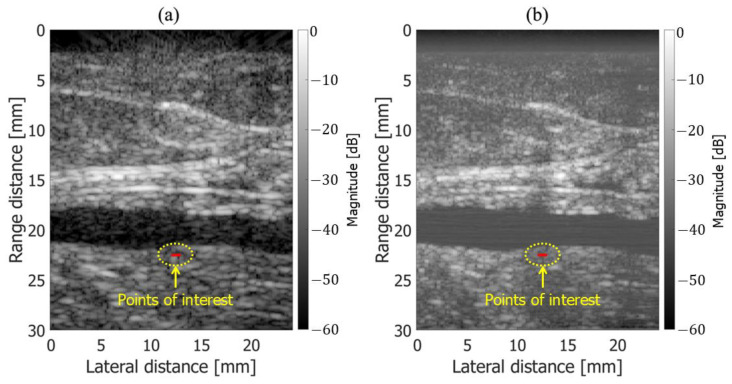
B-mode images with points of interest indicated by red lines. (**a**) Fundamental. (**b**) Harmonic.

**Figure 5:**
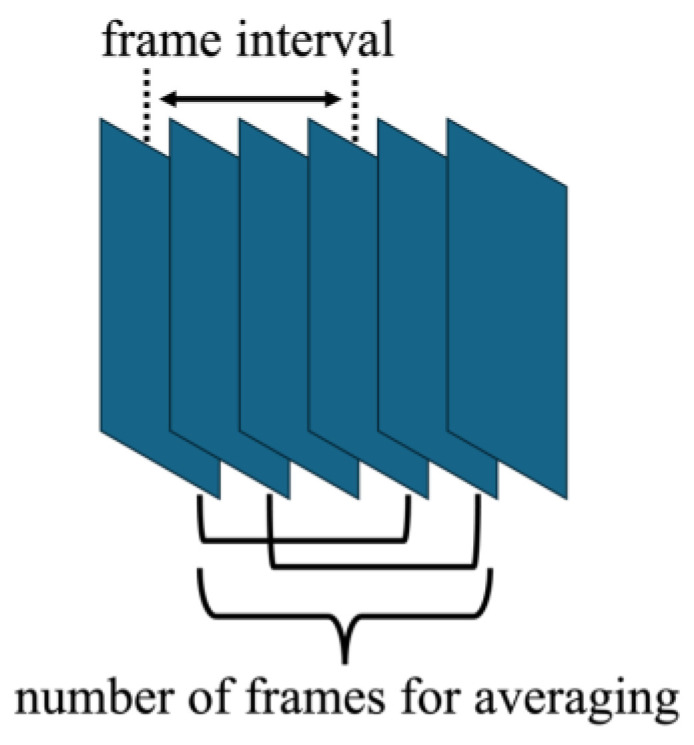
Illustration of frame averaging process in velocity estimation.

Figure 4a,b show B-mode images obtained using fundamental and harmonic imaging, respectively. Five points of interest (POIs) were placed horizontally above chicken thigh B along the red line in Figure 4. Velocity estimation was performed at each point under all experimental conditions. As illustrated in Figure 5, the frame interval used to compute the cross-spectrum in the 2D phase-sensitive motion estimator was varied from 1 to 4 to improve the stability of the velocity estimation results. Velocity estimation was performed both with and without frame averaging, where frame averaging involved computing the average of two separately estimated velocity values.

The root mean square error (RMSE) was used as an evaluation metric for the velocity estimation results. The RMSE of the estimated velocity was calculated as follows:

$${RMSE}_{\beta }=\sqrt{\frac{1}{n}\frac{{\sum {(\hat{v}}_{\beta }-v_{\beta })}^{2}}{v_{\beta }^{2}}},$$

where ${\hat{v}}_{\beta }$ is the estimated velocity, $v_{\beta }$ is the true velocity, n is the number of frames, and β is x or z. To reduce the influence of undesired vibrations of the jig caused by stage acceleration, the RMSE was consistently calculated during the stable movement period of the automated stage (0.2 to 0.8 s) across all experimental conditions.

### 2.3. In Vivo Experiment

**Figure 6:**
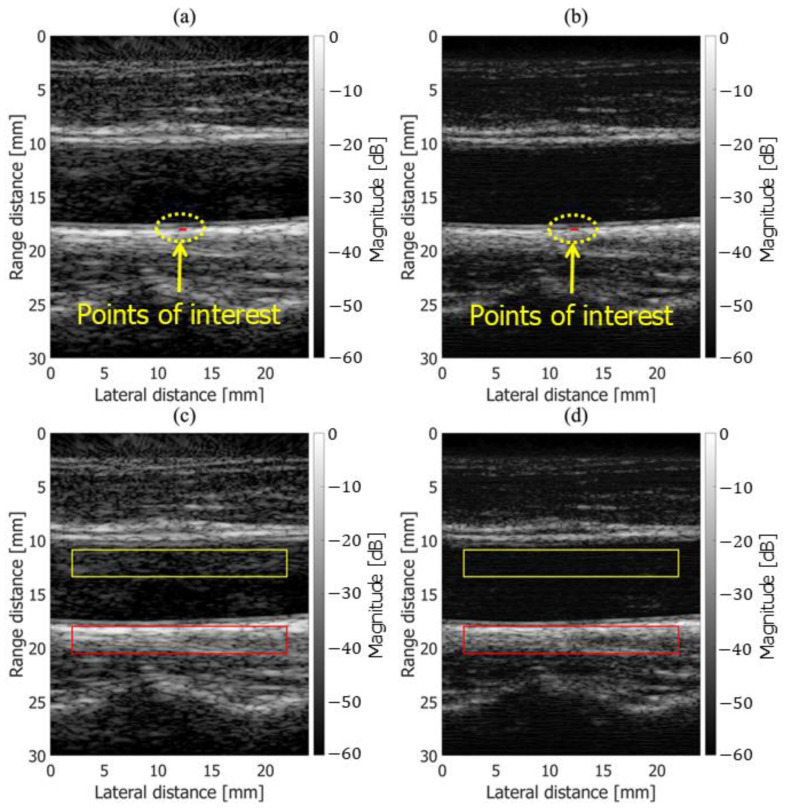
Examples of in vivo measurements. (**a**) Fundamental B-mode image with ROIs for velocity estimation. (**b**) Harmonic B-mode image with ROIs for velocity estimation. (**c**) Fundamental B-mode image with ROIs for contrast measurement. (**d**) Harmonic B-mode image with ROIs for contrast measurement.

In the in vivo measurement of the human carotid artery, ultrasound echo signals acquired from the common carotid artery wall of a healthy 51-year-old subject were analyzed. This study was approved by the institutional ethics committee and conducted with the subject’s informed consent. The measurement system used for the in vivo experiments was identical to that described in Section 2.2 for the basic experiments. Beamforming was performed with the speed of sound set to 1540 m/s. As shown in Figure 6a,b, regions of interest (ROIs), highlighted in red, were placed on the posterior wall of the carotid artery, and velocity estimation was performed using both fundamental and harmonic imaging. Each ROI contained 9 points arranged in a 3×3 grid along the axial and lateral directions, resulting in a size of [3×0.025=0.075 mm, 3×0.2=0.6 mm].

Additionally, as illustrated in Figure 6c,d, ROIs were placed within the vascular lumen and on the posterior wall to compare the contrast between fundamental and harmonic imaging in the B-mode images. The contrast was calculated using the following equation:

$$Contrast=20\log_{10}\frac{R\bar{O}I_{lumen}}{R\bar{O}I_{tissue}}\;\left(dB\right),$$

where $R\bar{O}I_{lumen}$ and $R\bar{O}I_{tissue}$ are the average echo amplitude values within the ROI of the vessel lumen and tissue, respectively.

## 3. Results

### 3.1. Basic Experimental Results

**Figure 7:**
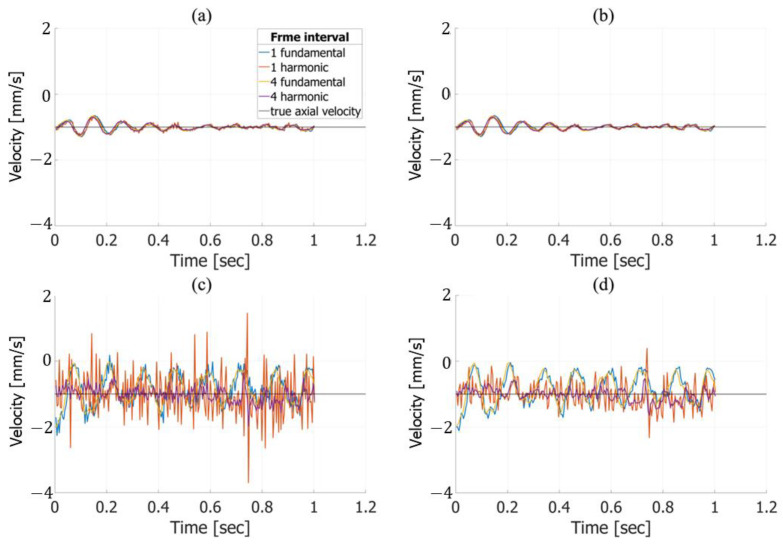
Velocity estimation results in the axial direction without (**a**) and with (**b**) frame averaging at a target velocity of −1.0 mm/s. Velocity estimation in the lateral direction without (**c**) and with (**d**) frame averaging.

Figure 7 illustrates the velocity estimation results for true axial and lateral velocities of −1.0 mm/s. For the axial direction, as shown in Figure 7a,b, the estimated velocities are similar regardless of the frame interval, frame averaging, or imaging method. In contrast, for the lateral direction, as depicted in Figure 7c,d, notable differences are observed between fundamental and harmonic imaging. The lateral velocity estimated using fundamental imaging exhibited oscillations, with minor variations depending on the frame interval. However, in harmonic imaging, changes in the frame interval significantly affected the estimation results, with larger intervals yielding estimates closer to the true value. Furthermore, as demonstrated in Figure 7d, applying frame averaging further improved the accuracy of the estimates, bringing them closer to the true value.

**Figure 8:**
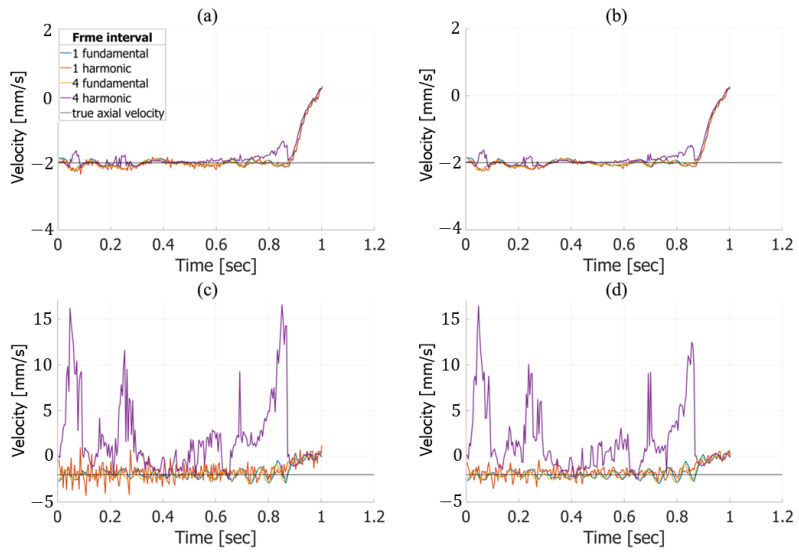
Velocity estimation results in the axial direction without (**a**) and with (**b**) frame averaging at a target velocity of −2.0 mm/s. Velocity estimation in the lateral direction without (**c**) and with (**d**) frame averaging.

Figure 8 presents the axial and lateral velocity estimation results for a true velocity of −2.0 mm/s. In Figure 8a,b, which show the axial velocity, at approximately 0.05, 0.2, and 0.8 s, only the results from harmonic imaging with a frame interval of 4 deviated from those obtained under other conditions. Figure 8c,d show the lateral velocity estimates using harmonic imaging with a frame interval of 1, which closely matched the true value. However, increasing the frame interval to 4 caused the estimates to deviate significantly from the true value. Moreover, the lateral velocity estimation results obtained using fundamental imaging exhibited noticeable oscillations.

Table 1 summarizes the RMSE values for axial and lateral velocity estimation, with and without frame averaging, for a true velocity of −1.0 mm/s. For the axial velocity, the RMSE decreased slightly with increasing frame intervals in both fundamental and harmonic imaging, although the changes were less than 1%. Applying frame averaging further reduced the RMSE for the axial velocity, but the improvement was also within 1% compared to the results without averaging. In the lateral direction under fundamental imaging, the difference in the RMSE across all conditions was, at most, approximately 6%, regardless of the frame interval or the number of frames averaged. In contrast, under harmonic imaging, results with a frame interval of 4 showed substantial improvements over those with a frame interval of 1. Specifically, without frame averaging, the RMSE improved by 35% when increasing the frame interval from 1 to 4, while, with frame averaging, the improvement was 14%. Additionally, applying frame averaging at a frame interval of 1 resulted in a 23% reduction in the RMSE compared to the non-averaged results. Table 2 presents the RMSE values for a true velocity of −2.0 mm/s in both the axial and lateral directions. For fundamental imaging, the effects of the frame interval and frame averaging on the RMSE were minimal, with the variations remaining within 2% in both directions. For harmonic imaging, the RMSE trends for frame intervals from 1 to 3 were consistent with those in Table 1. However, in the lateral direction, increasing the frame interval from 1 to 4 led to a significant deterioration in the RMSE, with an increase of approximately 120%, which was markedly greater than that observed for other intervals. These results suggest that the accuracy of lateral velocity estimation in harmonic imaging is highly sensitive to the frame interval setting.

### 3.2. In Vivo Results

**Figure 9:**
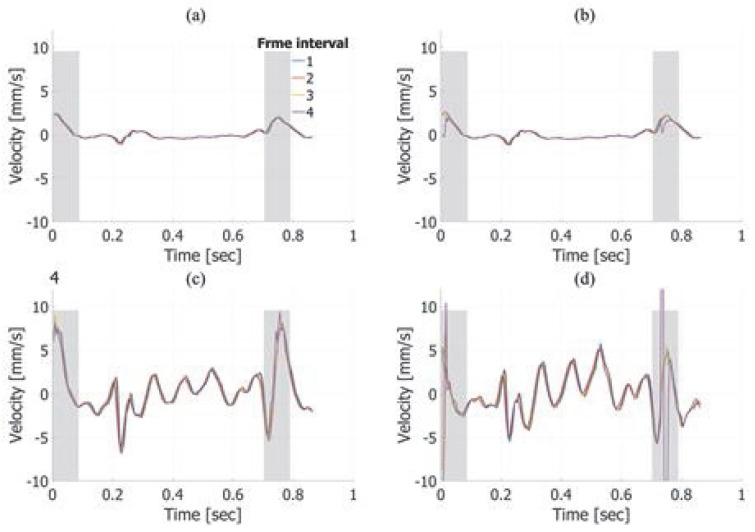
In vivo velocity estimation results. Axial velocity estimates obtained from fundamental (**a**) and harmonic (**b**) imaging without frame averaging. Lateral velocity estimates obtained from fundamental (**c**) and harmonic (**d**) imaging without frame averaging.

Figure 9 shows the temporal waveforms of the velocities averaged within the ROI under each condition, obtained from in vivo measurements of a healthy 51-year-old subject. These results were estimated without applying frame averaging. Focusing on the cardiac systole (indicated by the gray background), Figure 9a,b reveal the movement of the posterior vessel wall in the depth direction, corresponding to vessel expansion. Additionally, Figure 9c,d indicate that harmonic imaging with a frame interval of 4 produces waveforms that differ noticeably from those under other conditions—particularly in the lateral direction, where the estimated velocities exhibit significant variation. During cardiac diastole (indicated by the white background), only minor differences were observed, as shown across Figure 9a–d, likely due to variations in the frame interval. Furthermore, no substantial differences in axial or lateral velocity estimation were observed between fundamental and harmonic imaging.

**Figure 10:**
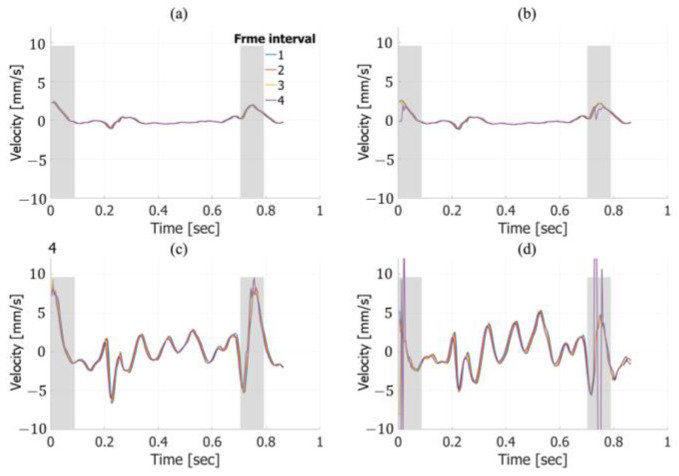
The same as in Figure 9, except with frame averaging applied. Axial velocity estimates obtained from fundamental (**a**) and harmonic (**b**) imaging. Lateral velocity estimates obtained from fundamental (**c**) and harmonic (**d**) imaging.

Figure 10 shows the temporal waveforms of the velocities averaged within the ROI under each condition. These results were estimated with frame averaging. The trends observed in Figure 10 are consistent with those in Figure 9, particularly in the lateral velocity estimates, which show significant variations under harmonic imaging with a frame interval of 4.

In the basic experiment, frame averaging improved the estimation accuracy in the lateral direction for harmonic imaging. However, in the in vivo experiment, the application of frame averaging did not lead to substantial changes in the lateral velocity estimation compared to the case without frame averaging.

**Figure 11:**
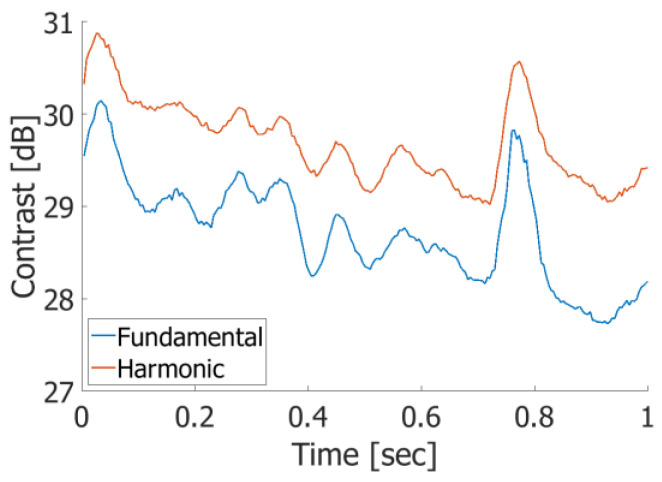
Contrasts between vessel lumen and surrounding tissue in fundamental and harmonic imaging.

As shown in Figure 11, harmonic imaging exhibits higher contrast values than fundamental imaging, with an average improvement of 0.96 dB, indicating a reduction in artifacts within the vessel lumen. These results suggest that the pulse inversion method employed in the proposed Tx–Rx sequence was successfully implemented, effectively enhancing the image quality by reducing intraluminal artifacts.

## 4. Discussion

The ultrasonic measurement of cardiovascular dynamics is valuable for the diagnosis of cardiovascular diseases. Color flow imaging is widely used in clinical settings for this purpose. In traditional Tx–Rx sequences for color flow imaging, multiple transmissions at the same aperture are required to enhance echoes from blood cells using a clutter filter. However, these multiple transmissions at each aperture position significantly reduce the imaging frame rate. To address this issue, high-frame-rate imaging using unfocused transmit beams was proposed. This approach enables frame rates exceeding several thousand frames per second and is beneficial for high-frame-rate blood flow imaging. However, it is difficult to combine this method with tissue harmonic imaging for high-contrast tissue visualization because the sound pressure of an unfocused transmit beam is significantly lower than that of a focused beam. Another technique to achieve high frame rates is the multi-line transmission imaging (MLTI) method using focused transmit beams. In this study, we propose a Tx–Rx sequence designed for velocity estimation that enables both high-frame-rate and high-contrast imaging simultaneously. Since the sequence employs focused transmit beams, it can be integrated with the pulse inversion (PI) method, as demonstrated in this study. The feasibility of the proposed sequence was validated through both in vitro and in vivo experiments. Table 3 summarizes the characteristics of representative imaging modes.

In the in vitro experiments, axial velocity estimation demonstrated consistent accuracy across different frame intervals and frame averaging conditions, as shown in Figure 7a,b and Figure 8a,b. For lateral velocity estimation, harmonic imaging yielded lower RMSE values than fundamental imaging at a frame interval of 3 with frame averaging, as indicated in Table 1 and Table 2. However, at a frame interval of 4, the RMSE for lateral velocity estimation in harmonic imaging increased significantly, indicating a marked decline in accuracy. This sharp rise in the RMSE highlights the sensitivity of harmonic imaging to the frame interval settings, particularly when the interval becomes excessively large. In contrast, fundamental imaging maintained consistent RMSE values across all frame intervals. When comparing the best velocity estimation performance between harmonic and fundamental imaging, harmonic imaging under optimal conditions—specifically, a frame interval of 3 with frame averaging—achieved superior RMSE values, as shown in Table 2. This suggests that harmonic imaging can provide more accurate velocity estimation when the parameters are carefully optimized. On the other hand, fundamental imaging exhibited stable performance across a broader range of frame intervals, indicating greater robustness under varying conditions.

Figure 7c and Figure 8c reveal oscillatory artifacts in lateral velocity estimation for fundamental imaging. The frequency of these oscillations increased from approximately 8 Hz to 16 Hz when the motion speed of the automated stage was changed from −1.0 mm/s to −2.0 mm/s, demonstrating a proportional relationship between the stage speed and the oscillation frequency. This proportionality suggests that the oscillations were caused by vibrations of the jig supporting the chicken thigh during the stage movement. While this mechanical resonance appears to be the primary cause, further investigation is required to rule out potential contributions from the velocity estimation algorithm itself.

The in vivo experiments assessed the performance of the proposed sequence in human carotid artery imaging. As shown in Figure 9a,b and Figure 10a,b, axial velocity estimation exhibited only minor differences between conditions with and without frame averaging, demonstrating its robustness in real-world settings. In contrast, the large difference observed between the results with and without frame averaging in the in vitro experiments is likely due to a lower signal-to-noise ratio, as the nonlinear response of water is weaker than that of biological tissue, resulting in smaller harmonic components compared to those in in vivo measurements.

For lateral velocity estimation, harmonic imaging exhibited a decline in estimation accuracy in regions with higher estimated velocities, as shown in Figure 10c,d. This tendency is consistent with the results from the in vitro experiments, particularly when the true velocity was −2.0 mm/s, as seen in Figure 8c,d. Moreover, in the in vitro experiments, the lateral estimation accuracy in harmonic imaging significantly deteriorated at a frame interval of 4 in regions with higher true velocities. This behavior, observed in both the in vitro and in vivo experiments, is likely caused by phase wrapping due to large displacements between two frames. These findings highlight a limitation of the velocity estimation algorithm in handling large motion, suggesting the need for optimization to maintain accuracy under high-velocity conditions. Additionally, studies aimed at mitigating the influence of this phenomenon would be beneficial.

Harmonic imaging provided improved imaging contrast, as shown in Figure 11. Although the numerical improvement in contrast was modest, it significantly reduced artifacts within the vascular lumen and enhanced the clarity of the vascular boundaries compared to fundamental imaging. These enhancements are particularly beneficial for diagnostic applications that require the clear visualization of vascular structures. The fundamental component is not perfectly canceled out when the target is in motion, resulting in well-known motion artifacts. Although this is a limitation of the PI method, such artifacts can be minimized by setting the interval between two transmissions to the pulse repetition time (PRT), as demonstrated in this study.

In this study, the number of simultaneous transmit (Tx) beams was set to four. The frame rate could be increased by using a greater number of simultaneous Tx beams. Some optimization may be possible to further improve the frame rate. However, the acquisition system used in this study does not allow the overlapping of simultaneous Tx apertures. Therefore, four simultaneous Tx beams represent the maximum achievable under the Tx aperture size shown to be feasible in our previous study [24]. The number of simultaneous Tx beams can be increased by reducing the Tx aperture size; however, this would result in decreased sound pressure. Although cross-talk artifacts tend to increase with a higher number of simultaneous Tx beams, a parameter optimization study—including the number of Tx beams, Tx and Rx aperture sizes, and apodization—may be beneficial to maximize the performance of the proposed sequence, assuming that an acquisition system that allows overlapping Tx apertures becomes available. In terms of hardware requirements, the number of transmit–receive channels increases with the number of simultaneous Tx beams. Similarly, the computational cost of generating beamformed RF signals also increases as the number of simultaneous Tx beams increases. Therefore, the proposed method may not be suitable for small-scale systems. Furthermore, the real-time capabilities of the proposed method should be investigated in future work.

In summary, the proposed sequence successfully integrates high-frame-rate velocity estimation with tissue harmonic imaging, enabling its application in scenarios that require both accurate velocity measurement and enhanced image quality. The proposed method is applicable to other probe types (e.g., phased-array or matrix probes) and other imaging modes (e.g., microvascular flow imaging) by adjusting the positioning and number of simultaneous Tx beams, as well as the frame averaging period.

## 5. Conclusions

This study proposed an extended Tx–Rx sequence that incorporates the pulse inversion method while maintaining a high frame rate of 231 Hz, building upon previous research. The basic experiments on velocity estimation demonstrated a practical level of accuracy, i.e., RMSE values of 3% (vertical) and 16% (horizontal), and the estimated velocity showed the motion of the vascular posterior wall with a typical cardiac cycle in the in vivo measurement. Moreover, harmonic imaging reduced artifacts and improved the contrast in the B-mode image by 0.96 dB. These results indicate that the proposed Tx–Rx sequence achieves both accurate vascular wall velocity estimation and artifact-reduced B-mode imaging simultaneously. In future studies, the effectiveness of the proposed method will also be evaluated in clinical cases involving atherosclerotic plaques. The influence of heterogeneous tissue on the proposed method is expected to be similar to that on other imaging modes. In particular, the change in the echo waveform between frames might be significant in cases of tissue with large deformation, e.g., soft tissue like lipids. Additionally, one limitation of the proposed method is the presence of motion artifacts inherent to the PI method, which may reduce the image contrast when imaging fast-moving targets such as cardiac walls. Further studies are necessary to adapt the proposed method for cardiac applications.

## Figures and Tables

**Table 1:** A value of −1 mm/s in both the axial and lateral directions, comparing fundamental and harmonic imaging under varying frame intervals and frame averaging conditions.

RMSE of Axial Velocity Estimation Results in Fundamental Imaging [%]		
frame interval	without frame averaging	with frame averaging
1	6.038	5.919
2	5.906	5.786
3	5.721	5.610
4	5.506	5.393
RMSE of axial velocity estimation results in harmonic imaging [%]		
frame interval	without frame averaging	with frame averaging
1	7.034	6.491
2	6.495	6.323
3	6.264	6.130
4	6.027	5.883
RMSE of lateral velocity estimation results in fundamental imaging [%]		
frame interval	without frame averaging	with frame averaging
1	46.571	44.743
2	43.959	43.455
3	42.531	42.019
4	41.056	40.518
RMSE of lateral velocity estimation results in harmonic imaging [%]		
frame interval	without frame averaging	with frame averaging
1	53.576	30.256
2	30.490	23.265
3	21.985	17.896
4	18.820	15.631

**Table 2:** A value of −2 mm/s in both the axial and lateral directions, comparing fundamental and harmonic imaging under varying frame intervals and frame averaging conditions.

RMSE of Axial Velocity Estimation Results in Fundamental Imaging [%]		
frame interval	without frame averaging	with frame averaging
1	3.519	3.516
2	3.494	3.493
3	3.496	3.455
4	3.476	3.408
RMSE of axial velocity estimation results in harmonic imaging [%]		
frame interval	without frame averaging	with frame averaging
1	3.563	3.164
2	3.161	3.062
3	3.047	2.994
4	3.802	3.684
RMSE of lateral velocity estimation results in fundamental imaging [%]		
frame interval	without frame averaging	with frame averaging
1	18.963	18.432
2	18.096	17.657
3	17.735	17.313
4	17.528	17.010
RMSE of lateral velocity estimation results in harmonic imaging [%]		
frame interval	without frame averaging	with frame averaging
1	33.152	22.361
2	21.872	18.883
3	17.669	16.002
4	153.275	148.791

**Table 3:** Comparison of characteristics of representative imaging modes.

	Single-Line Tx	MLTI	Unfocused Tx
Frame rate	<100 Hz	<1000 Hz	>1000 Hz
Tissue harmonic	Applicable	Applicable	N/A

## Data Availability

Data are contained within the article.

## References

[1] Nair A., Kuban B.D., Tuzcu E.M., Schoenhagen P., Nissen S.E., Vince D.G. (2002). Coronary Plaque Classification With Intravascular Ultrasound Radiofrequency Data Analysis. Circulation.

[2] Picano E., Paterni M. (2015). Ultrasound Tissue Characterization of Vulnerable Atherosclerotic Plaque. Int. J. Mol. Sci..

[3] Yamasaki Y., Kodama M., Nishizawa H., Sakamoto K., Matsuhisa M., Kajimoto Y., Kosugi K., Shimizu Y., Kawamori R., Hori M. (2000). Carotid intima-media thickness in Japanese type 2 diabetic subjects: Predictors of progression and relationship with incident coronary heart disease. Diab. Care..

[4] Kubozono T., Miyata M., Kawasoe S., Ojima S., Yoshifuku S., Miyahara H., Maenohara S., Ohishi M. (2017). High Pulse Wave Velocity Has a Strong Impact on Early Carotid Atherosclerosis in a Japanese General Male Population. Circ. J..

[5] Bots M.L., Hoes A.W., Koudstaal P.J., Hofman A., Grobbee D.E. (1997). Common carotid intima-media thickness and risk of stroke and myocardial infarction: The Rotterdam Study. Circulation.

[6] Nezu T., Hosomi N., Aoki S., Matsumoto M. (2016). Carotid Intima-Media Thickness for Atherosclerosis. J. Atheroscler. Thromb..

[7] Paul Y., Barthez D., Léveillé R., Peter V., Scrivani D. (1997). Side lobes and grating lobes artifacts in ultrasound imaging. Veter- Radiol. Ultrasound.

[8] Le H.T., Hangiandreou N., Timmerman R., Rice M.J., Smith W.B., Deitte L., Janelle G.M. (2016). Imaging Artifacts in Echocardiography. Anesth. Analg..

[9] Averkiou M., Roundhill D., Powers J. A new imaging technique based on the nonlinear properties of tissues. Proceedings of the 1997 IEEE Ultrasonics Symposium Proceedings. An International Symposium.

[10] Shapiro R.S., Wagreich J., Parsons R.B., Stancato-Pasik A., Yeh H.C., Lao R. (1998). Tissue harmonic imaging sonography: Evaluation of image quality compared with conventional sonography. Am. J. Roentgenol..

[11] Desser T.S., Jeffrey R.B., Lane M.J., Ralls P.W. (1999). Tissue harmonic imaging: Utility in abdominal and pelvic sonography. Clin. Ultrasound.

[12] Hann L.E., Bach A.M., Cramer L.D., Siegel D., Yoo H.H., Garcia R., Hann A.M.B.L.E., Shapiro R.S., Wagreich J., Parsons R.B. (1999). Hepatic sonography: Comparison of tissue harmonic and standard sonography techniques. Am. J. Roentgenol..

[13] Tranquart F., Grenier N., Eder V., Pourcelot L. (1999). Clinical use of ultrasound tissue harmonic imaging. Ultrasound Med. Biol..

[14] Rosenthal S.J., Jones P.H., Wetzel L.H. (2001). Phase Inversion Tissue Harmonic Sonographic Imaging. Am. J. Roentgenol..

[15] de Jong N., Bouakaz A., Cate F.J.T. (2002). Contrast harmonic imaging. Ultrasonics.

[16] Averkiou M.A., Bruce M.F., Powers J.E., Sheeran P.S., Burns P.N. (2020). Imaging Methods for Ultrasound Contrast Agents. Ultrasound Med. Biol..

[17] Brands P.J., Willigers J.M., Ledoux L.A., Reneman R.S., Hoeks A.P. (1998). A noninvasive method to estimate pulse wave velocity in arteries locally by means of ultrasound. Ultrasound Med. Biol..

[18] Rabben S.I., Stergiopulos N., Hellevik L.R., Smiseth O.A., Slørdahl S., Urheim S., Angelsen B. (2004). An ultrasound-based method for determining pulse wave velocity in superficial arteries. J. Biomech..

[19] Dunmire B., Beach K., Labs K.-H., Plett M., Strandness D. (2000). Cross-beam vector Doppler ultrasound for angle-independent velocity measurements. Ultrasound Med. Biol..

[20] Tortoli P., Dallai A., Boni E., Francalanci L., Ricci S. (2010). An Automatic Angle Tracking Procedure for Feasible Vector Doppler Blood Velocity Measurements. Ultrasound Med. Biol..

[21] Bohs L., Geiman B., Anderson M., Gebhart S., Trahey G. (2000). Speckle tracking for multi-dimensional flow estimation. Ultrasonics.

[22] Albinsson J., Brorsson S., Ahlgren Å.R., Cinthio M. (2014). Improved Tracking Performance of Lagrangian Block-Matching Methodologies Using Block Expansion in the Time Domain: In Silico, Phantom and in Vivo Evaluations. Ultrasound Med. Biol..

[23] Hasegawa H. (2016). Phase-Sensitive 2D Motion Estimators Using Frequency Spectra of Ultrasonic Echoes. Appl. Sci..

[24] Hasegawa H., Omura M., Nagaoka R., Saito K. (2022). Two-Dimensional Wavenumber Analysis Implemented in Ultrasonic Vector Doppler Method with Focused Transmit Beams. Sensors.

[25] Song P., Zhao H., Urban M.W., Manduca A., Pislaru S.V., Kinnick R.R., Pislaru C., Greenleaf J.F., Chen S. (2013). Improved Shear Wave Motion Detection Using Pulse-Inversion Harmonic Imaging With a Phased Array Transducer. IEEE Trans. Med Imaging.

[26] Duck F.A. (2002). Nonlinear acoustics in diagnostic ultrasound. Ultrasound Med. Biol..

[27] Carstensen E., Law W., McKay N., Muir T. (1980). Demonstration of nonlinear acoustical effects at biomedical frequencies and intensities. Ultrasound Med. Biol..

[28] Law W., Frizzell L., Dunn F. (1985). Determination of the nonlinearity parameter B/A of biological media. Ultrasound Med. Biol..

